# Trends in Incidence, Mortality, and Survival of Penile Cancer in the United States: A Population-Based Study

**DOI:** 10.3389/fonc.2022.891623

**Published:** 2022-06-17

**Authors:** Xinxi Deng, Yang Liu, Xiangpeng Zhan, Tao Chen, Ming Jiang, Xinhao Jiang, Luyao Chen, Bin Fu

**Affiliations:** ^1^ Department of Urology, Jiu Jiang No.1 People’s Hospital, Jiujiang, China; ^2^ Department of Urology, The First Affiliated Hospital of Nanchang University, Nanchang, China; ^3^ Department of Cardiology, The Second Affiliated Hospital of Nanchang University, Nanchang, China

**Keywords:** penile cancer, incidence, mortality, survival, SEER, epidemiology, trend

## Abstract

**Purpose:**

The aim of this study is to investigate the trends in incidence and mortality, and explore any change in survival of penile cancer in the United States.

**Methods:**

We obtained data from the Surveillance, Epidemiology, and End Results (SEER) database (2000–2018) utilizing the SEER Stat software. The joinpoint regression was used to analyze the secular trend of incidence and incidence-based mortality (IBM) stratified by age, race, and summary stage. The 5-year relative survival rate was also calculated.

**Result:**

The age-adjusted rates of penile cancer patients were 0.38 (0.37–0.39) and 0.21 (0.2–0.21) for overall incidence and IBM, respectively. The 5-year relative survival rates were 67.7%, 66.99%, and 65.67% for the calendar periods of 2000–2004, 2005–2009, and 2010–2014, respectively. No significant changes in incidence by era were observed from 2000 to 2018 [annual percentage change (APC) = 0.5%, *p* = 0.064]. The IBM rate of penile cancer showed an initial significant increase from 2000 to 2002 (APC = 78.6%, 95% CI, −1.7–224.6) followed by a deceleration rate of 4.6% (95% CI, 3.9–5.3) during 2002 to 2018. No significant improvement in 5-year relative survival was observed. The trends by age, race, and summary stage in incidence and IBM were significantly different.

**Conclusion:**

This study, using population-level data from the SEER database, showed an increasing trend in IBM and no significant improvement in the 5-year relative survival rate. Meanwhile, the incidence of penile cancer exhibited a relatively stable trend during the study period. These results might be due to the lack of significant progress in the treatment and management of penile cancer patients in the United States in recent decades. More efforts, like increasing awareness among the general population and doctors, and centralized management, might be needed in the future to improve the survival of this rare disease.

## Introduction

Penile cancer is a relatively rare neoplasm in Western countries, presenting an incidence rate of less than 1 per 100,000 men (1). However, a prominent geographic variation in incidence can be observed, and it may be due to different socioeconomic status, hygiene, religious, and cultural conditions (2, 3). For example, incidence rates in some developing countries like India (up to 2.3 per 100,000) and Eastern and Southern Africa (up to 2.7 per 100,000) were significantly higher than 1 per 100,000 men (1, 4). Brazil is one of the countries with the highest penile cancer incidence rates, which reached up to 3.3 per 100,000 based on record. A relatively higher mortality and a gradually increasing trend with an annual percent change (APC) of 1.4% are also reported in these countries during 1996 to 2010 (1, 5). Relative survival rates also showed a geographic variation between countries. For instance, the relative 5-year survival rate in Norway is 80%, while this value is only 62% in Finland from 1999 to 2003 (6). Risk factors confirmed to be associated with penile cancer include human papillomavirus (HPV) infection, smoking, circumcision status, and lower socioeconomic status (7–9). Although the exact pathogenesis is still unclear, some studies suggested that inflammation may play an essential role in tumor development or progression because tumors may likely arise from sites of penile infection and chronic irritation (10, 11).

Significant differences in incidence and mortality rate trends of penile cancer existed among different countries. For example, the trend in the incidence of penile cancer has been presented as increasing in Denmark during 1978–2008. However, in the United States, the trend of penile cancer incidence showed a significant decrease with a rate of 0.84, 0.69, and 0.58 per 100,000 for 1973 −1982, 1982−1992, and 1993−2002, respectively (12).

The trend of incidence rate and mortality rate of a disease can reflect the prevention, treatment, and management level of the disease, thereby deepening the understanding of disease and making recommendations for disease guidelines. As a developed country, America’s advanced medical technology and disease management strategy are often explored and used for reference by other countries. To our knowledge, there has been a lack of studies describing the trend of incidence, mortality, and survival of penile cancer in the United States over the past 10 years. In addition, a comparative study to explore the association between incidence, mortality, and survival rate has not been performed. This analysis, based on the Surveillance, Epidemiology, and End Results (SEER) database (2000–2018), aims to explore the up-to-date epidemiology of penile cancer in the United States. The trends in incidence, mortality, and survival of penile cancer by age, race, and summary stage are investigated according to the up-to-date information of epidemiology.

## Materials and Methods

### Data Sources and Study Population

We obtained penile cancer patients from the SEER Program of the National Cancer Institute (ID: 20420-Nov2020). Patients diagnosed with penile cancer as their first malignancy according to the list of *Site Recode the International Classification of Diseases for Oncology, Third Edition (ICD-O-3)* and cases who were coded with penis were enrolled in our study.

Incident cases were obtained from the database of incidence–SEER 18 registries of the US National Cancer Institute from 2000 to 2018, which collected data on cancer incidence and mortality involving approximately 26.4% of the U.S. population.

Incidence-based mortality (IBM) cases were obtained from the database of IBM–SEER 18 registries of the US National Cancer Institute from 2000 to 2018.

Survival cases were obtained from the database of incidence–SEER 18 registries of the U.S. National Cancer Institute from 2000 to 2014. We failed to acquire more data considering the 5-year relative survival rate was not recorded after 2014 in the SEER database. The study period was averagely divided into three time periods (2000–2004, 2005–2009, and 2010–2014) to observe prominent survival rate disparities.

### Outcomes and Descriptions

Three primary outcomes were calculated in this study: incidence, IBM, and 5-year relative survival rate. Incidence and IBM rates were adjusted to the 2000 U.S. standard population and calculated by 100,000 person-years. We calculated IBM rates as the number of all-cause death cases diagnosed with penile cancer among cases diagnosed over person-time at risk among people in areas of the SEER. In the registries of population-based SEER cancer, the incidence of individuals was linked to their mortality outcomes. It could calculate mortality rates by variables like the stage at diagnosis. This special mortality measure was defined as IBM (13, 14). Relative survival estimates were defined as the ratio of the observed survival of penile cancer patients and the expected survival of the underlying general population (15).

Then, we analyzed the annual percentage change (APC) of incidence and IBM rates stratified by age (15–44, 45–54, 55–64, 65–74, and 75+), race [White, Black, American/Indian/Alaska/Native (AIAN), and Asian/Pacific Islander (API)], and summary stage (localized, regional, and distant). Localized stage referred to an invasive neoplasm confined entirely to the penis (mainly including T_1-4_N_0_M_0_) and tumor staged as regional was defined as extending to surrounding organs, tissues, or regional lymph nodes (mainly including T_4_N_0_M_0_ or T_1-4_N_1-3_M_0_). Distant disease referred to the tumor that had spread to remote sites of the body (mainly including M_1_).

### Statistical Analysis

SEER_Stat version 8.3.2 was used to calculate incidence, mortality rates, and 5-year relative survival rate of penile cancer. Then, joinpoint regression was performed to identify the best-fitting log-linear regression model, which appropriately demonstrated the incidence and mortality rate trend by era. The National Cancer Institute’s Joinpoint Regression Program, Version 4.6.0.0, was utilized to calculate the APC and 95% confidence intervals (95% CIs) (16). The Joinpoint Regression software utilized *t*-tests to confirm whether statistical differences existed between APC and zero, and *p* < 0.05 was considered statistically significant. All statistical results were two-sided. Notably, we excluded the data not recorded from the joinpoint regression because no cases were reported at a certain year.

## Results

### Patient Characteristics

Finally, 6,397 patients diagnosed with penile cancer, who were from 18 SEER registries from 2000 to 2018, were enrolled in our study. **Table 1** demonstrates the characteristics of patients for incidence and IBM analysis. For all cases, the most common age group was 75+ years [2,132 (33.33%)], and White cases accounted for the most significant proportion in the study population [5,017 (83.55%)]. Compared to patients with other stages, patients with localized stage were more commonly seen [3,032 (47.4%)]. Of the eligible patients, 3,348 patients with penile cancer died during the study period. Of all the deaths, 1,399 (45.85%) patients were observed to be aged 75+ years, and 2,572 (84.3%) cases were White patients. A total of 1,495 (49%) patients who were recorded as dead were diagnosed with localized stage.

**Table 1:** Penile cancer incidence (2000–2018) and incidence-based mortality (2000–2018): the SEER-18 registry database.

Characteristic	Incidence		Incidence-based mortality	Rate (95% CI)
	Cases No. (%)	Rate (95% CI)	Deaths No. (%)	
**All patients (2000–2018)**	6,397 (100)	0.38 (0.37–0.39)	3,348 (100)	0.21 (0.2–0.21)
**Age, years**				
Overall (2000–2018)	6,397 (100)		3,051 (100)	
15–44	478 (7.47)	0.08 (0.07–0.08)	122 (4)	0.009 (0.007–0.01)
45–54	779 (12.18)	0.34 (0.32–0.37)	245 (8.03)	0.014 (0.013–0.016)
55–64	1,358 (21.23)	0.77 (0.73–0.81)	514 (16.85)	0.029 (0.027–0.032)
65–74	1,650 (25.79)	1.51 (1.44–1.59)	771 (25.27)	0.054 (0.05–0.058)
75+	2,132 (33.33)	2.31 (2.22–2.42)	1,399 (45.85)	0.094 (0.089–0.099)
**Race^1^**				
Overall (2000–2017)	6,005 (100)		3,051 (100)	
White	5,017 (83.55)	0.402 (0.391–0.413)	2,572 (84.3)	0.205 (0.198–0.214)
Black	597 (9.94)	0.401 (0.368–0.435)	328 (10.75)	0.241 (0.215–0.269)
AIAN	56 (0.92)	0.4 (0.296–0.525)	31 (1.02)	0.259 (0.172–0.37)
API	272 (4.53)	0.198 (0.175–0.223)	117 (3.83)	0.093 (0.077–0.112)
**Summary stage^2^**				
Overall (2000–2018)	6,397 (100)		3,051 (100)	
Localized	3,032 (47.4)	0.18 (0.18–0.19)	1,495 (49)	0.098 (0.093–0.104)
Regional	1,564 (24.45)	0.09 (0.09–0.1)	964 (31.6)	0.063 (0.063–0.067)
Distant	296 (4.63)	0.02 (0.02–0.02)	259 (8.49)	0.017 (0.015–0.019)

1: The limited number of patients whose race was unknown was excluded from further evaluation in the incidence and incidence-based mortality (IBM) (n = 63 and n = 3, respectively) analyses. Therefore, the percentages of patients of different races in the incidence and IBM analyses do not add up to 100%.

2: The limited number of patients whose summary was unknown was excluded from further evaluation in the incidence and incidence-based mortality (IBM) (n = 1505 and n = 332, respectively) analyses. Therefore, the percentages of patients of different races in the incidence and IBM analyses do not add up to 100%.

CI, confidence interval; SEER, Surveillance, Epidemiology, and End Results database. AIAN, American/Indian/Alaska/Native; API, Asian/Pacific Islander; NA, not applicable.

### Overall Incidence and Mortality Rates and Trends Over Time

**Figure 1:**
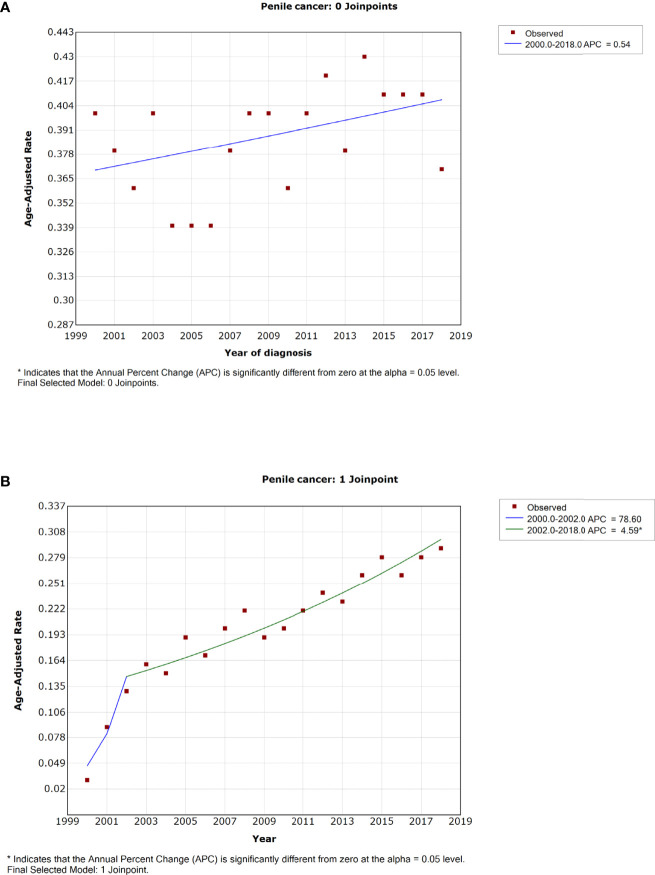
The overall trends in incidence **(A)** and incidence-based mortality **(B)** of penile cancer.

Of all study populations, the age-adjusted rates of penile cancer patients were 0.38 (0.37–0.39) and 0.21 (0.2–0.21) for incidence and IBM rate, respectively (**Table 1**). The incidence of penile cancer had no significant change from 2000 to 2018 (APC = 0.5%, 95% CI = −1.1–2.0; *p* = 0.064) (**Figure 1A** and **Table 2**). The IBM rate of penile cancer showed an initial significant increase from 2000 to 2002 (APC = 78.6%, 95% CI, −1.7–224.6) followed by a deceleration rate of 4.6% (95% CI, 3.9–5.3) during 2002 to 2018 (**Figure 1B** and **Table 2**).

**Table 2:** Trends in the incidence rates and incidence-based mortality of penile cancer (2000–2018): the SEER-18 registry database.

Characteristic	Incidence			Incidence-based mortality		
	Year	APC (95% CI)	*p*-value	Year	APC (95% CI)	*p*-value
**All patients**	2000–2018	0.5 (–1.1–2.0)	0.064	2000–2002	78.6 (−1.7–224.6)	0.052
				2002–2018	4.6 (3.9–5.3)	<0.001^※^
**Age, years**						
15–44	2000–2018	−0.5 (−2.0–1.1)	0.506	2000–2017	1.5 (−0.01–3.1)	0.054
45–54	2000–2018	1.9 (0.1–3.7)	0.043^※^	2000–2017	7.1 (3.4–10.8)	0.001^※^
55–64	2000–2018	0.9 (−1.7–3.5)	0.491	2000–2017	1.8 (0.6–3.2)	0.008^※^
65–74	2000–2018	−0.2 (−1.1–0.8)	0.741	2000–2002	99.1 (−0.2–297.1)	0.050
				2002–2017	4.3 (2.8–5.8)	<0.001^※^
75+	2000–2018	1.2 (0.5–1.9)	0.002^※^	2000–2017	4.8 (2.6–7.0)	<0.001^※^
**Race**						
White	2000–2017	0.7 (0.01–1.5)	0.044^※^	2000–2002	96.2 (24.0–210.4)	0.007^※^
				2002–2017	4.6 (3.6–5.5)	<0.001^※^
Black	2000–2017	0.4 (−0.8–1.6)	0.472	2000–2017	3.6 (0.6–6.6)	0.021^※^
AIAN	2000–2017	2.7 (−2.5–8.1)	0.289	2000–2017	1.7 (−0.5–4.0)	0.118
API	2000–2017	−0.7 (−3.1–1.7)	0.546	2000–2017	4.9 (0.6–9.4)	0.029^※^
**Summary stage**						
Localized	2000–2015	0.5 (−0.5–1.5)	0.313	2000–2005	29.6 (11.2–51.1)	0.003^※^
				2005–2017	2.7 (0.6–4.9)	0.015^※^
Regional	2000–2015	0.5 (−0.9–1.9)	0.459	2000–2002	94.8 (−1.0–283.5)	0.053
				2002–2015	4.7 (3.0–6.5)	<0.001^※^
				2015–2017	−32.5 (−49.9–−9.2)	0.014^※^
Distant	NA	NA	NA	2000–2017	1.8 (−1.0–4.6)	0.203

AIAN, American/Indian/Alaska/Native; API, Asian/Pacific Islander; NA, not applicable.

※: Statistical significance.

### Incidence and Mortality Rates and Trends by Age, Race, and Summary Stage

The penile cancer incidence rates were highest among cases aged over 75 years (2.31, 95% CI, 2.22–2.42), White patients (0.402, 95% CI, 0.391–0.413), and patients diagnosed with localized stage (0.18, 95% CI, 0.18–0.19) (**Table 1**). Similarly, the IBM rates of penile cancer were highest among patients aged 75+ years (0.094, 95% CI, 0.089–0.099), AIAN patients (0.259, 95% CI, 0.172–0.37), and patients with localized stage (0.098, 95% CI, 0.093–0.104) (**Table 1**).

**Figure 2:**
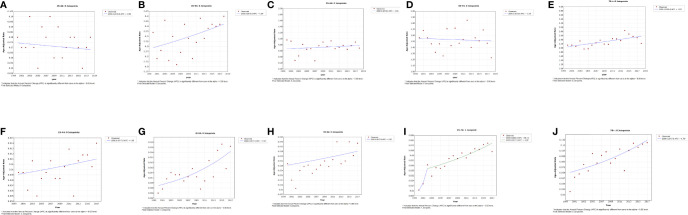
Trends in the annual incidence **(A–E)** and incidence-based mortality **(F–J)** of penile cancer in patients stratified by age at diagnosis.

The incidence rates among penile cancer patients in the age group of 45–54 and 75+ years exhibited a slight increase with an APC of 1.9% (95% CI, 0.1–3.7, *p* = 0.043) and 1.2% (95% CI, 0.5–1.9, *p* = 0.002), respectively, for the period of 2000–2018 (**Figures 2B, E** and **Table 2**). We did not obtain statistically significant trends in incidence rates in other age groups. For IBM rate analysis by age, patients diagnosed at ages 45–54, 55–64, and 75+ years exhibited an increasing trend at the rate of 7.1% (95% CI, 3.4–10.8, *p* = 0.001), 1.8% (95% CI, 0.6–3.2, *p* = 0.008), and 4.8% (95% CI, 2.6–7.0, *p* = 0.001), respectively, in 2000 to 2017 (**Figures 2G, H, J** and **Table 2**). In addition, for those aged 65–74 years, the trend of IBM presented a rapid initial increase (APC = 99.1%, 95% CI, −0.2–297.1) and then showed a deceleration for 2002–2017 (APC = 4.3%, 95% CI, 2.8–5.8, *p* < 0.001). (**Figure 2I** and **Table 2**).

**Figure 3:**
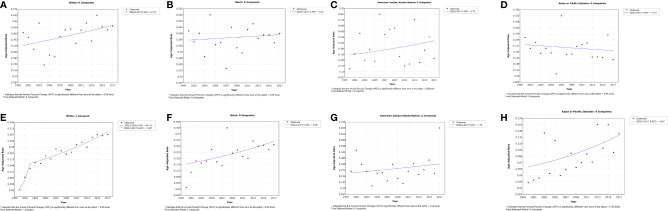
Trends in the annual incidence **(A–D)** and incidence-based mortality **(E–H)** of penile cancer in patients stratified by race.

A slightly increased incidence trend was observed from 2000 to 2017 with an APC of 0.7% (95% CI, 0.01–1.5, *p* = 0.044) among White penile cancer patients (**Figure 3A** and **Table 2**). No noticeable change in incidence was observed in other races. Of Black and API patients, the trend of IBM rates demonstrated a slowly rising trend with an APC of 4.6% (95% CI, 3.6–5.5, *p* < 0.001) and 4.9% (95% CI, 0.6–9.4, *p* = 0.029), respectively (**Figures 3F, H** and **Table 2**). The IBM rate in White patients increased sharply at the initial time of 2000 to 2002 (APC = 96.2%, 95% CI 24.0–210.4, *p* = 0.007), and the increasing trend had slowed down in 2002 (APC = 4.6%, 95% CI 3.6–5.5, *p* < 0.001). (**Figure 3E** and **Table 2**).

**Figure 4:**
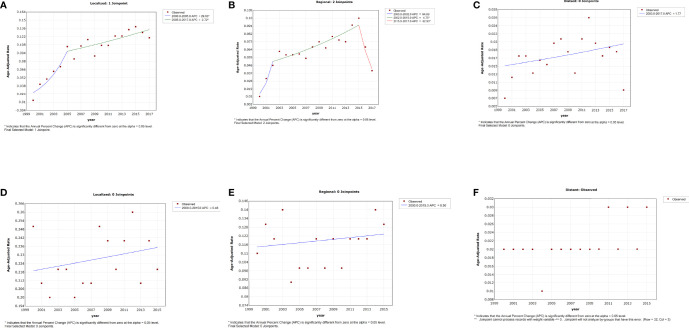
Trends in the annual incidence **(D–F)** and incidence-based mortality **(A–C)** of penile cancer in patients stratified by summary stage.

No significant changes were observed in the incidence trend by summary stage from 2000 to 2015. We failed to obtain a best-fitting line and APC for patients with distant stage due to the relatively low incidence and the lack of regular variation (**Figure 4F**). Of patients diagnosed with localized stage, the trend of IBM rates showed an initial prominent increase during 2000 to 2005 (APC = 29.6%, 95% CI = 11.2–51.1, *p* = 0.003), followed by a deceleration thereafter (APC = 2.7%, 95% CI, 0.6–4.9, *p* = 0.015) (**Figure 4A** and **Table 2**). For patients with regional stage, a continuous increasing IBM rate was observed from 2002 to 2015 (APC = 4.7%, 95% CI, 3.0–6.5; *p* < 0.001), but a steep decline in the trend of IBM rates was exhibited after 2015 (APC = −32.5%, 95% CI, −49.9–−9.2, *p* = 0.014) (**Figure 4B** and **Table 2**).

### Trend of the 5-Year Relative Survival Rate of Penile Cancer

The overall 5-year relative survival rates showed a slight decrease with a rate of 67.7% (SE = 1.76%), 66.99% (SE = 1.7%), and 65.67% (SE = 1.66%) for the time periods 2000–2004, 2005–2009, and 2010–2014, respectively (**Table 3**). However, this change was not statistically significant (*p* = 0.12). The descending trend in relative survival rates was observed in White and Black patients, and it was relatively prominent in Black patients (change −8.08%, *p* < 0.001) (**Table 3**). For patients diagnosed with localized stage, the 5-year relative survival rates exhibited an increasing trend with a rate of 76.6% (SE = 2.2%), 79.57% (SE = 2.11%), and 81.55% (SE = 2.1%) for time periods 2000–2004, 2005–2009, and 2010–2014, respectively (change 4.95%, *p* < 0.001) (**Table 3**). For API, the 5-year relative survival rates increased from 72.62% in 2000–2004 to 91.52% in 2005–2009, and then dropped to 65.72% in 2010–2014. Similar trends were observed in patients diagnosed with regional stage and diagnosed at 15–44, 45–54, and 75+ years. However, none of these trends was regular and easily to explain.

**Table 3:** Five-year relative survival rate of penile cancer patients by race, stage, and age.

Characteristic	Year diagnosed Change^※^									
		2000–2004			2005–2009			2010–2014		
	*N*	5-year rate	SE	*N*	5-year rate	SE	*N*	5-year rate	SE	
**Overall**	1,175	67.7%	1.76%	1,187	66.99%	1.7%	1,379	65.67%	1.66%	−2.03%
**Race**										
White	996	67.18%	1.91%	1,007	66%	1.86%	1,133	65.63%	1.85%	−1.55%
Black	122	67.66%	5.64%	105	65.88%	5.61%	140	59.58%	4.9%	−8.08%
API	46	72.62%	7.68%	52	91.52%	4.61%	71	65.72%	6.37%	−6.87%
**Stage**										
Localized	687	76.6%	2.2%	703	79.57%	2.11%	800	81.55%	2.1%	4.95%
Regional	371	59.74%	3.21%	367	62.03%	3.11%	422	52.69%	2.91%	−7.05%
Distant	59	17.29%	5.51%	61	14.75%	4.83%	96	15.56%	4.03%	−1.73%
**Age**										
15–44	145	77.61%	3.75%	121	78.51%	4%	123	73.34%	4.44%	−4.27%
45–54	201	69.93%	3.46%	175	81.31%	3.22%	184	69.42%	3.69%	−0.54%
55–64	305	72.7%	2.9%	278	69.58%	3.07%	342	69.74%	2.86%	−2.96%
65–74	353	67.62%	3.09%	291	65.82%	3.36%	351	71.11%	3.16%	3.49%
75+	391	56.83%	3.92%	322	63.77%	4.2%	379	54.86%	4.17%	−1.97%

Change**^※^**: in the 5-year relative survival between 2000–2004 and 2010–2014, in % units.

5-year rate: 5-year relative survival rate.

N, number of patients; SE, standard error; API, Asian/Pacific Islander.

## Discussion

This study comprehensively explored the trend of incidence, IBM, and 5-year relative survival rate of penile cancer in the United States during 2000–2018, and further examined the trend by stratifying age, race, and tumor stage. There were no significant changes in the trend of incidence of penile cancer from 2000 to 2018. However, we found that the IBM rate of penile cancer significantly increased and that there was no significant improvement in the 5-year relative survival rate over the study period.

The incidence rate of penile cancer, at 0.38 per 100,000 over 2000 to 2018, was relatively lower than the result from a previous study based on the SEER database (12). They found an incidence rate of 0.84, 0.69, and 0.58 per 100,000 for the calendar periods 1973−1982, 1982−1992, and 1993−2002, respectively, and the data were collected from 9 SEER registries, which cover approximately 9.4% of the U.S. population. Compared to the previous incidence, the incidence in this study had still decreased. However, considering the geographical variation, we should seriously explain this discrepancy considering that our data came from 18 registries.

There is a relatively big difference between the trend in the incidence rate of different countries. For instance, the trend in the incidence of penile cancer was increasing for Denmark over 1978–2008 and England over 1979–2009 (17, 18), whereas this tendency was inverse in Finland during 1971–1995 and the United States during 1973–2002 (19, 20). In this study, we found a stable trend in the incidence of penile cancer in the United States during 2000–2018, although we obtained a slight upward best-fitting line (*p* > 0.05, **Figure 1A**). Although we observed a slight increase in incidence rate for patients aged 45–54 and 75+ years and White patients, the extent of this change was quite small. Previous studies usually explain the decreasing incidence rate with improved sanitation, declining smoking rate, and newborn male circumcision (21–23). For example, several data-based studies suggested that the rate of male circumcision ranged from 42% to 80% in the United States, and the procedure is commonly performed during the newborn period (24). The available evidence proved that male circumcision had special benefits in preventing urinary tract infection, HIV infection, the transmission of some sexually transmitted infections, and penile cancer (25). A relatively higher rate of male circumcision was considered a protective factor for penile cancer, and it might be a crucial reason for the stable incidence in the United States. Another notable reason was chronic inflammation, which was considered as a significant pathogenic pathway of penile cancer (25–28). A relatively perfect healthcare system and universal sex education might account for the lower rate of chronic inflammation than those in developing countries. These results showed that the prevention of penile cancer in the United States had a good performance.

There were relatively few studies focusing on the trend of penile cancer mortality. A retrospective study, whose data were from the Netherlands during 1989–2006, suggested a slight decrease in mortality (11, 29). Similarly, a decrease in mortality was also observed in England for 1979–2009 (30). Nevertheless, we found a prominent increase in the IBM rate in the United States for the period of 2000–2018. Interestingly, a rapid increase of IBM was observed at the initial period of 2000–2002 (APC = 78.6%), but it failed to obtain a statistically significant *p*-value due to the relatively short study period. Similarly, of patients aged 65–74 years, White cases, and patients with regional stage, we also observed a sharp increase in IBM in the initial period of 2000–2002 (**Figures 2I**, **3A**, **4B**). In addition, we also did not observe any improvement in the 5-year relative survival rate of penile cancer in the United States. The phenomenon of no significant improvement in the 5-year survival rate and increased mortality of penile cancer might be due to the lack of significant progress in the treatment and management of penile cancer (31).

A likely explanation for these results was difficult to make. A recent study suggested that penile sparing surgery had been increasingly adopted, and no prominent differences in survival were observed between patients undergoing sparing and complete surgery (32). This improved surgical approach might lead to a better quality of life. Still, its contribution to high-risk patients, especially those with positive lymph nodes and distant metastasis, was not remarkable. In the past two decades, the most significant progress in the treatment of penile cancer was treating primary lesions, modified lymphadenectomy, and identifying and treating occult regional lymph node metastasis with the help of sentinel lymph node biopsy (SNB) (33, 34). About 80% of patients with one or two lymph nodes involved can be cured by lymphadenectomy. Even patients with pelvic lymph node involvement can still be cured by surgery.

The main goal of SNB was to reduce mortality and improve survival in clinical lymph node-negative (cN0) patients. Reported data showed that about 20% to 25% of the cN0 penile cancer patients had occult lymph node metastases at diagnosis, and early surgical resection of these occult lymph nodes could obtain better survival than those with clinically apparent nodes (35). The introduction of SNB might thus have improved survival, especially those with occult lymph nodes. An unpublished study from the Netherlands does show that cancer-specific survival in cN0 patients had improved since the introduction of SNB. However, we did not observe an improvement in 5-year relative survival, and even an increase in mortality in patients with penile cancer was obtained in this study. This result might account for the relatively low referral rate to hospitals specializing in the treatment of penile cancer, or the improvement of penile cancer treatment had not been fully implemented in hospitals.

For the management of penile cancer, several European countries have centralized management of penile cancer. The interval between diagnosis and treatment was significantly shortened, and compliance with the guidelines for patients with penile cancer was improved through this method (36, 37). Notably, the major delay in diagnosis of penile cancer was the time between the first symptom and diagnostic confirmation considering that patients and doctors might misinterpret the symptoms of penile cancer as condyloma, benign phimosis, or benign skin disease. This centralized management strategy could shorten this interval. In addition, this strategy was proved to work in improving survival and reducing mortality in the long run. Verhoeven et al. compared the 5-year relative survival rate of penile cancer patients between Europe and the United States over 1985–2007, and they found an increase from 65% to 70% and a decrease from 72% to 63% in the 5-year relative survival rate for Europe and the United States, respectively (38). For Norway and Denmark, the 5-year relative survival increased from 61% to 80% and 63% to 74%, respectively (6). However, the United States had not fully adopted this centralized management system, which might be an important explanation for the condition.

Another possible explanation for this result was that the main population of penile cancer patients was aging. For example, previous studies showed that the most common age of penile cancer patients was between 50 and 70 years (29, 39). However, patients aged 75+ years were the main population age group in our study. A higher proportion of elderly patients might lead to higher mortality and poor survival in penile cancer patients.

This is the first study that comprehensively explored the epidemiology of a rare disease from incidence, IBM, and 5-year relative survival for the period of 2000 to 2018 in the United States. The condition of penile cancer patients seemed to not have a noticeable improvement and progression considering the increasing IBM and the lack of significant change in the 5-year relative survival rate. Multiple comprehensive factors like changes in treatment and demographics, increase in exposure to HPV, and variation of cancer should be considered when interpreting results (22, 23, 40).

Several limitations should be noted when interpreting the results of this study. First, we selected a data list that collected epidemic information of approximately 26.4% of the U.S. population. Meanwhile, a relatively shorter study period was also chosen compared to previous similar studies. In addition, except for the low case numbers resulting in high standard errors of incidence, IBM, and survival estimates, essential pieces of information such as HPV infection, smoking, diagnosis, and follow-up treatment were not obtained in the SEER database. Finally, similar to the limitations of most epidemiological studies, our study has revealed a phenomenon in a period but cannot provide a definite explanation for the condition (6, 23, 38, 41). Therefore, more evidence was needed to explain these results.

## Conclusion

The current study, using population-level data from the SEER database, provides valuable data on penile cancer. It shows an increasing trend in IBM and no significant improvement in the 5-year relative survival rate among penile cancer patients for the period of 2000 to 2018 in the United States. Meanwhile, the incidence of penile cancer exhibited a relatively stable trend during the study period. These results indicate the lack of significant progress in the treatment and management of penile cancer patients in the United States in recent decades. More efforts, like increasing awareness among the general population and doctors, and centralized management, may be needed in the future to improve the survival of this rare disease.

## Data Availability Statement

The original contributions presented in the study are included in the article/supplementary material. Further inquiries can be directed to the corresponding authors.

## Ethics Statement

The data from SEER is publicly available and de-identified. This study was approved by the institution of the First Affiliated Hospital of Nanchang University. No informed consent was needed.

## Author Contributions

XD, YL and XZ contributed equally to this work. All authors listed have made a substantial, direct, and intellectual contribution to the work and approved it for publication.

## Funding

This study was supported by the National Natural Science Foundation of China (Grant Nos. 81560419, 81960512, and 81760457) and Jiangxi Provincial “Double Thousand Plan” Fund Project (Grant No. jxsq2019201027).

## Conflict of Interest

The authors declare that the research was conducted in the absence of any commercial or financial relationships that could be construed as a potential conflict of interest.

## Publisher’s Note

All claims expressed in this article are solely those of the authors and do not necessarily represent those of their affiliated organizations, or those of the publisher, the editors and the reviewers. Any product that may be evaluated in this article, or claim that may be made by its manufacturer, is not guaranteed or endorsed by the publisher.
